# Evaluation of Antibiotic Resistance and Biofilm Production among Clinical Strain Isolated from Medical Devices

**DOI:** 10.1155/2021/9033278

**Published:** 2021-08-14

**Authors:** Veronica Folliero, Gianluigi Franci, Federica Dell'Annunziata, Rosa Giugliano, Francesco Foglia, Rossella Sperlongano, Anna De Filippis, Emiliana Finamore, Massimiliano Galdiero

**Affiliations:** ^1^Department of Experimental Medicine, University of Campania “Luigi Vanvitelli”, Naples 80138, Italy; ^2^Department of Medicine Surgery and Dentistry “Scuola Medica Salernitana”, University of Salerno, Fisciano 84081, Italy; ^3^U.O.C. of Virology and Microbiology, A.O.U. University of Campania “Luigi Vanvitelli”, Naples 80138, Italy

## Abstract

Microbial biofilms pose a serious threat to patients requiring medical devices (MDs). Prolonged periods of implantation carry a high risk of device-related infections (DRIs). Patients with DRIs often have negative outcomes following the failure of antibiotic treatment. Resistant DRIs are mainly due to the MDs contamination by bacteria producing biofilm. The present study aimed to detect biofilm formation among MD bacterial isolates and to explore their antibiotic resistance profile. The study was conducted on 76 MDs, collected at University Hospital of Campania “Luigi Vanvitelli,” between October 2019 and September 2020. Identification of isolates and antibiotic susceptibility testing were performed using Matrix Assisted Laser Desorption Ionization-Time of Flight Mass Spectrometry (MALDI-TOF MS) and Phoenix Becton Dickinson, respectively. Biofilm-forming abilities were assessed using the tissue culture plate (TCP) method. Among the 94 MDs isolated strains, 42.7% were Gram-positive, 40.3% Gram-negative, and 17% Candida species. Among 78 bacterial strains, 43.6% were non-biofilm producers while 56.4% produced biofilms. All biofilm producing isolates were sensitive to a limited spectrum of antibiotic classes. All moderate and strong biofilm producers and 81% of weak biofilm producers were Multidrug Resistance (MDR) strains. In contrast, among non-biofilm producers, only 11.8% were classified as MDR strains. Our results highlighted that Sulfamides and Glycopeptides for the major Gram-positive strains and Fluoroquinolones, Carbapenems, and Aminoglycosides for the most represented Gram-negative isolates could be the most suitable therapeutic choice for most biofilm-DRIs.

## 1. Introduction

Biofilm is a structured bacterial community, enclosed in a self-produced polymeric matrix and adhered to biotic or abiotic surfaces [1]. Compared to their planktonic counterparts, biofilm-associated bacteria exhibit greater resistance to antibiotic agents [2]. This increased antibiotic resistance is mainly due to the limited diffusion of drugs through the biofilm matrix and to physiological changes in bacteria due to the environmental conditions featuring the biofilm [3]. Furthermore, the physical proximity of the cells in the biofilm favors the acquisition of resistance through genetically transmissible elements [4]. Several pieces of evidence revealed a positive correlation between biofilm formation and the development of antibiotic resistance [5]. Abidi et al. showed that biofilm production was higher among MDR *Pseudomonas aeruginosa* (*P. aeruginosa*) strains than non-MDR strains [6]. Moreover, Qayoom et al. found that the *Acinetobacter baumannii* (*A. baumannii*) MDR produced more biofilm than the non-MDR ones [7]. Regarding *Staphylococcus aureus* (*S. aureus*), Manandhar et al. associated biofilm production with Methicillin resistance [8, 9].

Biofilms readily develop in MDs, widely used in almost all areas of medicine for diagnostic and therapeutic processes and for the management of critically ill patients [1]. Prolonged use of MDs in hospitalized patients leads to a high risk of bacterial and fungal infections, defined as DRIs [10]. DRIs occur in patients with MDs in use for at least 48 hours before the infection begins [11, 12]. These infectious diseases are feared, causing a significant morbidity and mortality and an increase of healthcare costs [13]. According to data from the Centers for Disease Control and Prevention (CDC), of nearly 2 million nosocomial infections, 50–70% were related to MD [14]. Mortality attributed to DRIs is strongly associated with the type of device, ranging from <5% for devices such as Foley catheters to >25% for central venous catheters (CVCs) [14]. The three most common DRIs are centerline-associated bloodstream infection (CRBSIs), ventilator-associated pneumonia, and Foley catheter-associated urinary tract infection [15]. Among these infections, CRBSIs are highly prevalent [16]. Among these infections, CRBSIs are highly prevalent, detecting approximately 80,000 cases annually in US intensive care units. Gram-positive and Gram-negative bacteria and fungi contribute to the emergence of DRIs. The most frequently encountered strains include coagulase-negative staphylococci (CoNS), *Enterococcus faecalis* (*E. faecalis*), *Klebsiella pneumoniae* (*K. pneumoniae*), *A. baumannii*, *P. aeruginosa*, and *Candida albicans* [7]. Among these, *S. aureus* and *Staphylococcus epidermidis* (*S. epidermidis*) are estimated to cause approximately 50–70% of catheter biofilm infections [17, 18]. The main concern about DRIs is represented by the difficulty in their eradication, due to the high antibiotic resistance of MD biofilm-associated bacteria [19]. Antibiotic therapy often unsuitable for the treatment of these infections, due to the resistant nature of the biofilm, promotes the development of serious clinical complications among DRI patients [4]. This scenario highlights the need to better understand the device-related bacteria, in order to improve the management and treatment of DRIs [20, 21]. Therefore, the present study aimed to detect the presence of biofilm-forming isolates from different MDs and to explore their antibiotic resistance pattern. Knowledge of the main MD strains and related antibiotics susceptibility profile is essential to allow the optimal choice of antibiotic therapy for DRIs.

## 2. Materials and Methods

### 2.1. Sample Collection

In this study 76 MDs were collected at the University Hospital of Campania “Luigi Vanvitelli” (Naples, Italy) from October 2019 and September 2020. Devices included 65 CVCs, 8 Foley catheters, 2 nephrostomy tubes, and 1 abdominal drain tube (Table 1). The MDs were delivered to the bacteriology laboratory and then processed.

### 2.2. MDs Culture and Isolate Identifications

Each MD was cut into two pieces, using sterile forceps. One piece was rolled onto the surface of a Columbia agar plate supplemented with 5% sheep blood (Oxoid, Cheshire, UK) in the presence of CO_2_. The other part was placed in 10 mL of Brain Heart Infusion broth and incubated for 24 hours. Broth was inoculated on CNA blood, MacConkey, Sabouraud Glucose, and Chocolate agar medium (Oxoid, Cheshire, UK). All plates were assessed after O/N incubation at 37°C and further incubated for 48 hours if growth was not obvious. In positive cases, identification and antimicrobial sensitivity tests were performed. Bacterial identifications were conducted through MALDI-TOF MS (Bruker Dal-tonics, Germany). A colony from a culture agar plate was plotted on a MSP 96 MALDI-TOF (Bruker Dal-tonics, Germany), treated with 1 *μ*L of matrix solution (saturated solution of alpha-cyano-4-hydroxycinnamic acid in 50% of acetonitrile and 2.5% of trifluoroacetic acid) (Bruker Dal-tonics, Germany) and dried for 2 minutes. The obtained spectra were imported into MALDI BioTyper 3.0 software (Bruker Dal-tonics, Bremen, Germany) and evaluated through standard pattern matching with respect to the main spectra. A score greater than or equal to 2.0 was associated with species identification [22, 23].

### 2.3. Antimicrobial Susceptibility Testing

The Phoenix BD (Becton Dickinson, United States) was used to confirm the identification of strains obtained via MALDI-TOF MS and to perform antibiotic susceptibility tests. Briefly, the identification broth (ID) was inoculated with pure bacterial colonies and adjusted to 0.5 McFarland, using a Phoenix spectrophotometer. Phoenix AST broth was complemented with a drop of Phoenix AST indicator and, after that, a 25 *µ*l volume of bacterial suspension was added. ID and AST broth were loaded into the Phoenix panels, which were deposited in the Phoenix device. The results were interpreted using Epicenter software version 7.22 A (Becton Dickinson Diagnostic Systems, USA) after 16 hours of incubation [24]. The tested antibiotics in this study were ampicillin, amoxicillin/clavulanic acid, amikacin, cefotaxime, cefuroxime, fosfomycin, gentamicin, imipenem, levofloxacin, trimethoprim/sulfamethoxazole, tobramycin, piperacillin, piperacillin/tazobactam, cefotaxime, clindamycin, oxacillin, and daptomycin. Interpretative breakpoints for susceptibility and resistance were in accordance with EUCAST guidelines 2021 [25]. Resistance greater than or equal to 3 antibiotic classes defined the bacteria as MDR strain [26].

#### 2.3.1. Biofilm Formation Assays

Biofilm formation was assessed by TCP method. Shortly, bacterial cells were inoculated in Luria Bertani (LB) broth supplemented with 1% glucose and incubated for 24 h at 37°C. After incubation, the cultures were adjusted to OD_600 nm_ of 0.2 (∼10^8^ CFU/mL) with fresh LB medium with glucose. From standardized bacterial suspensions, a volume of 100 *μ*L of culture was inoculated into wells of a flat bottom (96-well, Thermo Fisher Scientific, Massachusetts, USA) and incubated at 37°C for 24 hours, statically. LB without cells were used to check sterility of media. *S. aureus* ATCC 6538 and *S. aureus* ATCC 25923 were used as negative and positive controls for biofilm production, respectively. After incubation, the biofilm was washed twice with 1X phosphate buffered saline (1X PBS) to remove free floating planktonic bacteria and air-dried. The wells were stained with 0.1% Crystal Violet (CV) (Sigma-Aldrich, St. Louis, MO) for 40 min at room temperature and washed three times with 1X PBS to remove excess dye. The remaining CV was solubilized by incubating with 95% ethanol for 20 min under orbital shaking at room temperature. Biofilm biomass was detected by measuring the absorbance at 570 nm, using microplate reader TECAN (Sunrise, Delaware, USA). Biofilm production was classified as negative, weak, moderate, and strong based on the cutoff value, calculated according to the following formula, using the optical density (OD) values [27]:

ODcutoff = ODavg of negative control + (3 × standard deviation of ODs of negative control).

The used criteria were as follows:

- OD ≤ ODcutoff = Non-biofilm former
- ODcutoff < OD ≤ 2 × ODcutoff = Weak biofilm former
- 2 × ODcutoff < OD ≤ 4 × ODcutoff = Moderate biofilm former
- OD > 4 × ODcutoff = Strong biofilm former

All assays were performed in triplicate.

### 2.4. Biofilms Visualization by Scanning Electron Microscopy (SEM)

The biofilms were grown on stainless steel coupons placed in a 12-well polypropylene microplate (Microtech, Naples, Italy). A volume of 1 mL of bacterial suspension at a density of 1 × 10^8^ CFU/mL was added to each selected well and the plate was statically incubated for 24 hours at 37°C. After that, the coupons were washed with 1X PBS, air-dried, and treated with plasma for 0, 3, and 30 min. Samples were fixed in glutaraldehyde (2.5% *v*/*v*) for 30 minutes and then dehydrated with cold solutions of ethanol at increasing concentrations (30, 50, 70, 90, 95, and 100% *v*/*v*), each for 20 minutes. All samples were dried in a critical point desiccator (Emitech K850, Kent, UK). Then, about 15–20 nm gold spray coating was performed with the Balzers SCD 030 (New York, USA) and the images were achieved using the Supra 40 ZEISS (EHT = 5.00 kV, WD = 22 mm, detector in the lens) (Berlin, Germany) [28].

### 2.5. Statistical Analysis

Statistical analysis was conducted using the IMB SPSS software (version 22.0; IBM SPSS Inc., New York, USA). Descriptive statistics were performed for medical device distribution and collected isolates. The antibiotic susceptibility profile of bacterial strains was expressed in percentage. Fisher's test was used to evaluate the relation between two groups of categorical variables. A *p* value of greater than or equal to 0.05 was considered statistically significant [29, 30].

## 3. Results

### 3.1. Prevalence of Microbial Contamination of MDs

**Figure 1:**
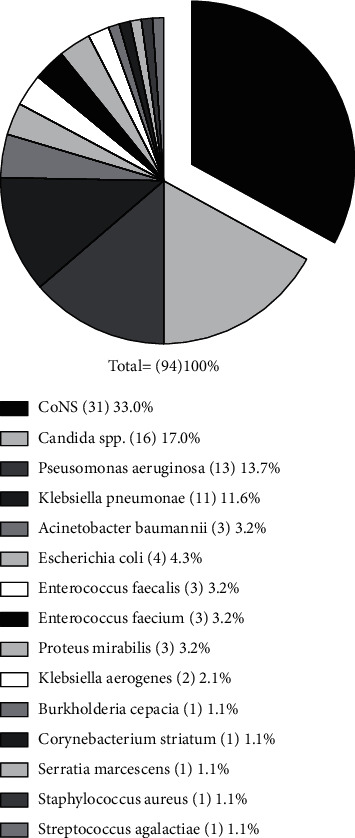
Prevalence of bacterial and yeast strains isolated from medical devices.

**Figure 2:**
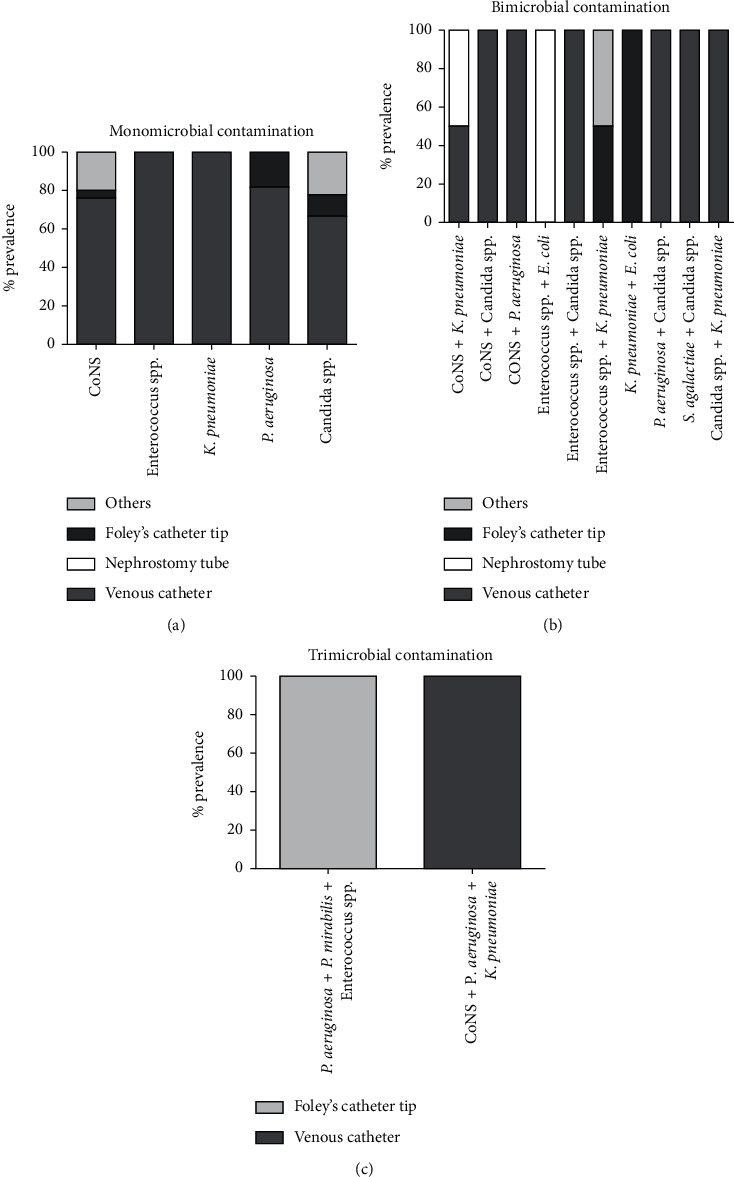
Monomicrobial (a), bimicrobial (b), and trimicrobial (c) contamination of the medical devices studied.

From 76 MDs, 94 strains were isolated, of which 42.7% (40) were Gram-positive, 40.3% (38) were Gram-negative, and 17% (16) were Candida species. Among Gram-positive bacteria, CoNS strains were the most frequently detected isolate (33%), followed by *E. faecalis* (3.2%) and *Enterococcus faecium* (*E. faecium*) (3.2%), *Corynebacterium striatum* (*C. striatum*) (1.1%), *Staphylococcus aureus* (*S. aureus*) (1.1%), and *Streptococcus agalactiae* (*S. agalactiae*) (1.1%). On the other hand, most representative Gram-negative bacterial strains were *P. aeruginosa* (13.7%), succeeded by *K. pneumoniae* (11.6%), *Escherichia coli* (*E. coli*) (4.3%), *A. baumannii* (3.2%), *Proteus mirabilis* (*P. mirabilis*) (3.2%), *Klebsiella aerogenes* (K. aerogenes) (3.2%), *Burkholderia cepacia* (*B. cepacia*) (1.1%), and *Serratia marcescens* (*S. marcescens*) (1.1%) (Figure 1).  Table 2 shows that CVCs were mainly colonized by CoNS strains (42.0%), followed by Candida species (21.7%), *P. aeruginosa* (14.5%), and *K. pneumoniae* (10.1%), *A. baumannii* (4.4%), *P. mirabilis* (2.9%), *Enterococcus* species (2.9%), and *E. coli* (1.5%). Bacteria isolated in the nephrostomy tubes were *Enterococcus* spp. (25%), CoNS strains (25%), *K. pneumoniae* (25%), and *E. coli* (25%). In the abdominal drain tube *K. pneumoniae* (50%) and *Enterococcus* spp. were detected. Moreover, Foley's catheter tips were mainly colonized by *P. aeruginosa* (25%), succeeded by *Enterococcus* species (16.7%), *K. pneumoniae* (16.7%), and *E. coli* (16.7%), CoNS strains (8.3%), *P. mirabilis* (8.3%), and Candida species (8.3%). The current analysis revealed that some devices were colonized by more than one microorganism. The monomicrobial contaminations accounted for 77.6%, while polymicrobial growths were detected in 22.4% of devices with 19.8 and 2.6% for bimicrobial and trimicrobial contaminations, respectively. Fungal/bacterial and bacterial/bacterial copresence were each associated with a prevalence of 41.2 and 58.8%. The devices subject to the greatest polymicrobial contamination were CVCs, showing the copresence of Candida species with CoNS, *Enterococcus* strains, *P. aeruginosa*, *S. agalactiae*, and *K. pneumoniae.* In contrast, in the trimicrobial contaminations only bacterial species were present (Figure 2).

### 3.2. Prevalence of Antimicrobial Resistance among Bacteria Isolated from MDs

**Figure 3:**
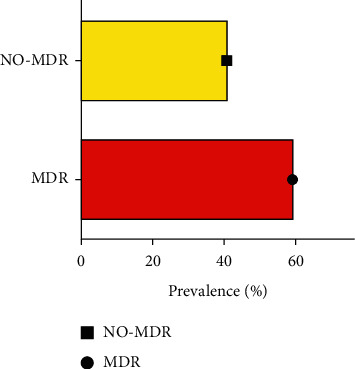
Distribution of MDR and NO-MDR bacterial strains isolated from medical devices (*p* value < 0.05).

In this study, the antimicrobial resistance profiles of *S. aureus*, CoNS, *C. striatum*, *S. agalactiae*, *E. faecium*, *E. faecalis*, *A. baumannii*, *E. coli*, *K. pneumoniae*, *P. aeruginosa*, *K. aerogenes*, *B. cepacia*, and *P. mirabilis* were evaluated. The antimicrobial resistance patterns of Gram-positive and Gram-negative strains are shown in Tables 3 and 4, respectively. Among Gram-positive bacteria, CoNS strains were the most frequent, exhibiting more than 60% resistance rate to several tested antibiotics: ampicillin, ciprofloxacin, erythromycin, moxifloxacin, oxacillin, penicillin g, and rifampicin. In contrast, resistance rates to phosphomycin, teicoplanin, tigecycline, and vancomycin were less than 20%. Of the 31 CoNS isolates, 74.2% showed methicillin resistance phenotype and 9% macrolide-lincosamide-streptogramin B resistance phenotype. The Gram-positive strain with the highest susceptibility rate was *E. faecalis*; indeed, only 33.3% were resistant to Gentamicin (Table 3). Regarding Gram-negative bacteria, *P. aeruginosa* was more encountered but *A. baumannii* represented the most resistant strain, showing 100% of resistance to all tested antibiotics, except for colistin. About *P. aeruginosa*, this strain had 61% resistance to fosfomycin and less than 15.8% to cefepime, ciprofloxacin, tigecycline, gentamicin, meropenem, piperacillin/tazobactam, and colistin. Of the 13 *P. aeruginosa* isolated, 7.7% had an extended spectrum *β*-lactamases-producing phenotype. Critical antibiotic resistance profile was associated with *K. pneumoniae* strains that exhibited resistance greater than 60% to ampicillin, cefepime, amoxicillin/clavulanic acid, ertapenem, ceftazidime, cefotaxime, levofloxacin, meropenem, piperacillin, and piperacillin/tazobactam (Table 4). Among *K. pneumoniae* isolates, 54.5% produced extended spectrum beta-lactamases, 9% showed macrolide-lincosamide-streptogramin B resistance phenotype, and 45.5% were bla_*KPC*_-type carbapenemase producers. The analysis of the antibiotic susceptibility profiles of the isolated bacteria revealed that the non-MDR strains were 41.1%, while the MDR ones were 58.9% (*p* value < 0.05) (Figure 3).

### 3.3. Detection of Biofilm Production by Bacteria Isolated from MDs

**Figure 4:**
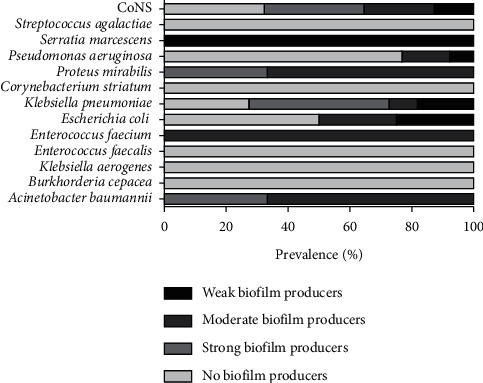
Biofilm formation among MD isolates.

In this study, strains isolated from different MDs were evaluated for their ability to produce biofilms. Of 78 bacterial strains tested, 43.6% (34) were non-biofilm producers while 56.4% (44) produced biofilms. *S. agalactiae*, *C. striatum*, *E. faecalis*, *K. aerogenes*, and *B. cepacia* were all non-biofilm producers. On the other side, all *A. baumannii* produced biofilms, of which 33.3% were strong biofilm producers and 66.7% moderate biofilm producers. The same biofilm production trend was detected for *P. mirabilis*. One identified strain of *S. marcescens* weakly produced biofilms. For *E. coli*, only 50% produced biofilms, of which 25% were moderate biofilm producers and the other 25% were weak biofilm producers. The 76.9% rate of *P. aeruginosa* was non-biofilm producers while 23.1% were biofilm producers; of the latter, 15.4 and 7.7% were moderate and weak biofilm producers, respectively. About *K. pneumoniae*, 72.7% were biofilms producers in which 18.2, 9.1, and 45.4% were weak, moderate, and strong producers, respectively. Among CoNS strains, 66.7% produced biofilms, of which 32.3, 22.6, and 12.8% were strong, moderate, and weak biofilm producers, respectively (Figure 4).

#### 3.3.1. Biofilm Production and Antimicrobial Resistance in Bacterial Strains Isolated from MDs

**Figure 5:**
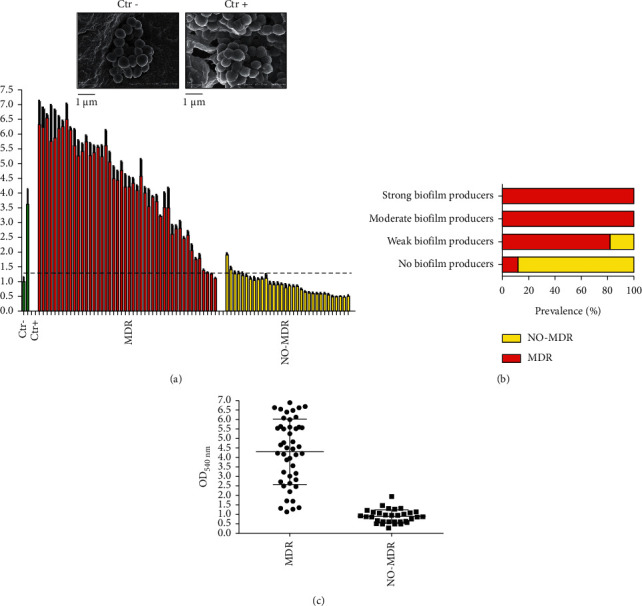
Biofilm formation analysis of MD isolates. (a) Biofilm production level of MDR, red bars, and NO-MDR, yellow bars. The green bars indicate negative and positive controls, also represented by SEM. Dashed line represents the cutoff value. (b) Prevalence of MDR and NO-MDR strains among the different biofilm productions. (c) Distribution of biofilm formation with indicated resistance phenotypes.

**Figure 6:**
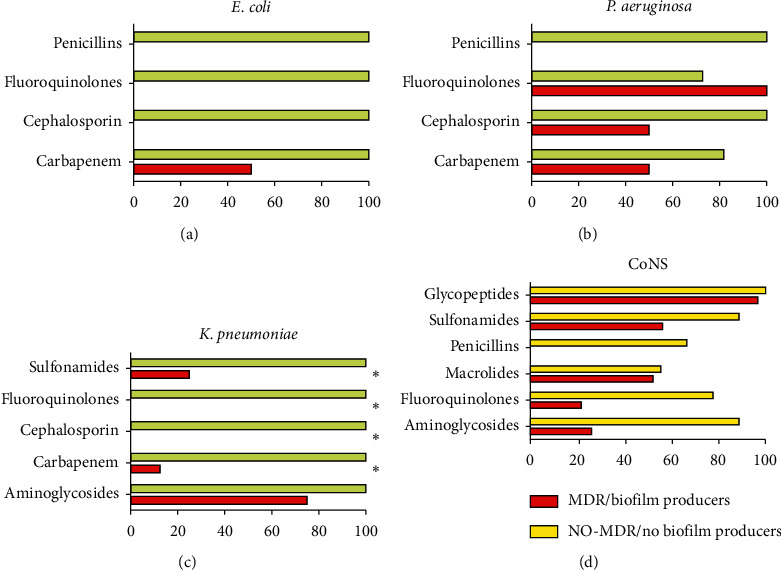
Susceptibility rate to different classes of antibiotics in *E. coli* (a), *P. aeruginosa* (b), *K. pneumoniae* (c), and CoNS (d) between biofilm and non-biofilm producers. ^*∗*^*p* value< 0.05.

The ability to secrete the biofilm matrix of MDR and non-MDR strains was assessed through the TCP method. The OD_570 nm_ values, as a measure of biofilm mass, related to MDR, non-MDR, and control strains, were reported in Figures 5(a) and 5(c). The absorbance values were 1.0 ± 0.156 and 3.62 ± 0.517 for the negative and positive standard strains for biofilm production, respectively. For clinical isolates, these values ranged from 6.326 ± 0.80 and 0.4718 ± 0.308. The OD_570 nm_ values of 54.6% (44) strains exceeded the calculated cutoff value; of these, 95.5% (42) were MDR strains and 4.5% (2) exhibited a non-MDR phenotype. In contrast, 43.6% (34) strains were associated with absorbance values below the cutoff value; of the latter 88.2% were non-MDR and 11.8% were MDR strains (*p* value < 0.05). Among the non-biofilm producing strains, 11.8% were MDR and 88.2% non-MDR isolates. Weak biofilm producers consisted of 81.8% MDR and 18.2% non-MDR strains. Instead, all moderate and strong biofilm producers resulted as MDR strains (Figure 5(b)). These data underlined the positive correlation between biofilm formation and resistance phenotype, main cause of DRI treatment problems. The sensitivity profiles to penicillins, fluoroquinolones, cephalosporins, carbapenems, sulfonamides, aminoglycosides, macrolides, and glycopeptides of *E. coli*, *P. aeruginosa*, *K. pneumoniae*, and CoNS biofilm and non-biofilm producers were evaluated and compared (Figure 6). All biofilm producing isolates had a high rate of resistance to the analyzed antibiotic classes. Among *E. coli*, all biofilm producing strains were resistant to penicillins, fluoroquinolones, and cephalosporins, while only 50% showed sensitivity to Carbapenems. In contrast, the non-biofilm producing counterparts were susceptible to all antibiotic classes tested (Figure 6(a)). Dwelling on the biofilm producing *P. aeruginosa* strains all exhibited resistance to Penicillins, unlike non-biofilm producers. For cephalosporins and carbapenems, biofilm-producing *P. aeruginosa* showed 50% susceptibility rates, whereas the non-biofilm producing counterparts were 100 and 81.8% sensitive, respectively. For Fluoroquinolones, a susceptibility of 100 and 72.7% was detected for producing and nonproducing biofilm, respectively (Figure 6(b)). Biofilm-producing *K. pneumoniae* exhibited susceptibility levels of 75, 25, and 12.5% to aminoglycosides, sulfonamides, and carbapenems, respectively. In contrast, all biofilm producing *K. pneumoniae* strains were resistant to Fluoroquinolones and Cephalosporins. Sensitivity of 100% to all tested antibiotic classes was found for non-biofilm producing strains. Significant variations in sensitivity levels of biofilm producers and nonproducers to sulfonamides, carbapenems, fluoroquinolones, and cephalosporins were detected (*p* value < 0.05) (Figure 6(c)). Regarding biofilm producing CoNS strains, they exhibited 100% resistance to penicillins and susceptibility rates of 96.8, 21.7, 26, 52.5, and 56.2% to glycopeptides, fluoroquinolones, aminoglycosides, macrolides, and sulfonamides, respectively. In contrast, all non-biofilm producing CoNS strains were susceptible to glycopeptides, while the sensitivity rates ranging from 55.5 to 88.8% were found for fluoroquinolones, aminoglycosides, macrolides, and sulfonamides. Significant differences were encountered between the sensitivity levels of biofilm producing and non-biofilm producing strains to aminoglycosides, fluoroquinolones, and penicillins (*p* value < 0.05) (Figure 6(d)).

## 4. Discussion

Extensive and prolonged use of MDs is associated with a significant risk of infectious complications that prolong hospitalization and raise healthcare costs [10]. Most patients with DRIs have a negative outcome, following antibiotic therapy failure. Resistant DRIs are mainly due to complex characteristics of bacterial biofilms, such as the shielding effect of the biofilm matrix which limits the penetration of antibiotic, the physical proximity of bacterial cells which promotes the exchange of resistance gene elements, and the slow cell growth rate [31]. Therefore, the present study defines the main pathogens that contaminate MDs, their propensity to form biofilms, and the related patterns of antibiotics susceptibility, to improve the management and treatment of DRIs. To conduct this analysis, 94 strains were isolated from CVC, Foley catheters, nephrostomy, and abdominal drain tube over a period of approximately 1 year. Among these isolates, 42.7% were Gram-positive, 40.3% were Gram-negative, and 17% were Candida species. About the Gram-negative bacteria, *P. aeruginosa*, *K. pneumoniae*, and *E. coli* were the most prominent, while, among Gram-positive bacteria, CoNS species represented the most isolated strains. The mainly isolated MDs strains belonged to normal commensal flora or were of nosocomial origin [32]. The highest incidence of Gram-positive strains (44.9%) was detected in the CVC samples, in accordance with other studies. Sohail and Latif revealed that 64% of CVCs were colonized by Gram-positive bacteria, 26% by Gram-negative bacteria, and 10% by Candida species [33]. Likewise, Lombardi et al. reported that 54% of CVCs were colonized by Gram-positive bacteria [34]. Our data reported that CoNS strains contributed most to the contamination of CVCs, as reported by Lombardi et al. [34]. In contrast the study conducted in Pakistan identified *S. aureus* as common pathogen found in these devices (39%) [33]. A wider prevalence of Gram-negative strains (66.7%) was detected in the Foley catheters, with a higher incidence of *P. aeruginosa* (25%). Almalki and Varghese detected 89% of Gram-negative bacteria on the same devices, of which *E. coli* was the most frequently encountered (26%) [35].

Analysis of the antibiotic susceptibility profiles of all isolated strains allowed classifying the bacteria in MDR and NO-MDR strains. Our data detected 59.2 and 40.8% of MDR and NO-MDR strains, respectively. These strains were evaluated for their propensity to produce biofilms. Of the 78 bacterial strains investigated, 56.4% were biofilm producers. The major producers were *A. baumannii* (100%), *S. marcescens* (100%), and *P. mirabilis* (100%), *K. pneumoniae* (72.7%), CoNS strains (67.7%), and *E. coli* (50%). Similar data were reported in the study of Revdiwala et al., in which the largest biofilm producers isolated from MDs were CoNS strains (88.9%), *K. pneumoniae* (100%), *E. coli* (68.8%), and *A. baumannii* (95.3%) [36]. The population showing biofilm production contained a high percentage of isolated MDR. Indeed, all moderate and strong biofilm producers and 81.8% of weak biofilm producers were MDR strains. Several studies revealed a positive correlation between biofilm formation and the development of antibiotic resistance [37]. In particular, Abidi et al. showed that biofilm production was higher among MDR *P. aeruginosa* strains than in non-MDR strains [6]. Moreover, Amin et al. found that the *A. baumannii* MDR strains produced more biofilm than the non-MDR ones [7]. The association between the MDR phenotype and biofilm production underlined the need to better investigate the resistance profiles of biofilm producing strains. Bacterial resistance patterns showed that *E. coli* biofilm producing strains exhibited 100% resistance to Penicillins, Fluoroquinolones, and Cephalosporins and 50% sensitivity to Carbapenems. Our data showed that Carbapenems could be efficient for the antibiotic treatment of DRIs caused by *E. coli* biofilm producing strains. In agreement with our study, Penicillin resistance of approximately 90% was reported in a study conducted in Nepal by Neupane et al. In contrast to our data, they noted resistance rates of 58.3 and 50% related to Fluoroquinolones and Cephalosporins [38]. Regarding *P. aeruginosa*, the biofilm producing strains were all resistant to Penicillins, while they were sensitive to cephalosporins (50%), carbapenems (50%), and fluoroquinolones (100%). The sensitivity levels of these biofilm producing strains indicated fluoroquinolones as a potential treatment for DRIs due to *P. aeruginosa.* Our data agreed with the study of Abidi et al., reporting that *P. aeruginosa* biofilm producing strains exhibited lowest resistance against fluoroquinolones and cephalosporins [6]. Concerning *K. pneumoniae*, all biofilm producing strains were resistant to fluoroquinolones and cephalosporins. Susceptibility rates below 75% were recorded for sulfonamides, carbapenems, and aminoglycosides. The high sensitivity rates to aminoglycosides indicated that it could be used for treatment of *K. pneumoniae* biofilm producing strains. Our data was in contrast with a study conducted in Indonesia, which attributed a resistance rate higher than 70.83 to Aminoglycosides and lower than 1.40% to carbapenems [39]. About CoNS biofilm producing strains, they showed susceptibility levels higher than 56.2% to sulfonamides and glycopeptides and lower than 52.2% to macrolides, fluoroquinolones, and aminoglycosides. Due to the increased susceptibility levels to glycopeptides and sulfonamides, they could be selected as a potential treatment for DRIs caused by CoNS biofilm producing strains. Similar data were obtained from a study conducted by Shresthe et al. that reported 100, 78.3, and 3.2% of CoNS biofilm producing strains resistant to penicillins, fluoroquinolones, and glycopeptides, respectively. In contrast, they listed resistance levels of 66, 80, and 50% for macrolides, sulfonamides, and aminoglycosides, respectively [40]. The reported low levels of antibiotic susceptibility reflect a worrying reality associated with the DRIs. Several studies reported similar conditions in other contexts, highlighting the generalized spread of MDs-related resistances [41]. Currently, there are no guidelines for the clinicians to treat DRIs, although they are associated with untreatable cases [42]. We suggest that empirical antibiotic treatment should be based on localized epidemiological trend data. Our study provides information on the current situation in our University Hospital, to define novel guidelines for the correct treatment of DRIs.

## 5. Conclusions

In the present study, the worrying prevalence of MDR biofilm producing strains represents a serious challenge to clinicians in the treatment and care of hospitalized patients. Antibiotics belonging to the class of glycopeptides and sulfonamides for CoNS strains and fluoroquinolones, carbapenems, and aminoglycosides for *P. aeruginosa*, *E. coli*, and *K. pneumoniae* isolates were found to be more effective for most biofilm producing strains. A better understanding of the biofilm producing strains isolated from MDs and related antibiotic resistance profiles could help define a more effective treatment plan to improve patient management and stimulate the scientific community to search for novel treatment strategies to combat this real threat [43].

## Figures and Tables

**Table 1:** Medical devices distribution.

MDs	*n* (%)
**Venous catheter (CVC)**	65 (85, 6)
**Nephrostomy tube**	2 (2, 6)
**Abdominal drain tube**	1 (1, 3)
**Foley's catheter tip**	8 (10, 5)
Total	76 (100)

**Table 2:** Main bacterial and yeast isolates from different medical devices.

	*Enterococcus* spp. (*n*) %	CoNS (*n*) %	*K. pneumoniae* (*n*) %	*P. aeruginosa* (*n*) %	*E. coli* (*n*) %	*A. baumannii* (*n*) %	*P. mirabilis* (*n*) %	Candida spp. (*n*) %
**Venous catheter**	(2) 2.9	(29) 42.0	(7) 10.1	(10) 14.5	(1) 1.5	(3) 4.4	(2) 2.9	(15) 21.7
**Nephrostomy tube**	(1) 25.0	(1) 25.0	(1) 25.0	(0) 0.0	(1) 25.0	(0) 0.0	(0) 0.0	(0) 0.0
**Abdominal drain tube**	(1) 50.0	(0) 0.0	(1) 50.0	(0) 0.0	(0) 0.0	(0) 0.0	(0) 0.0	(0) 0.0
**Foley's catheter tip**	(2) 16.7	(1) 8.3	(2) 16.7	(3) 25	(2) 16.7	(0) 0.0	(1) 8.3	(1) 8.3

**Table 3:** Antibiotic resistance profile of Gram-positive bacteria.

Antibiotic resistance profile of Gram-positive bacteria (%)																	
	AMP	CIP	DA	DAP	E	FOS	GM	LNZ	MXF	OX	TEC	TET	TIG	PG	RIF	VA	SXT
***S. aureus***	100	0	0	0	0	0	0	0	0	0	0	0	0	100	100	0	0
**CoNS**	100	61.3	38.7	0	61.3	19.4	58.1	0	61.3	74.2	9.7	41.9	9.7	100	100	3.2	58.1
***C. striatum***	—	—	100	—	—	—	—	0	0	—	—	0	—	100	100	0	—
***S. agalactiae***	0	0	100	—	100	—	0	0	0	—	0	0	—	0	—	0	—
***E. faecium***	100	—	—	—	—	—	66.6	0	—	—	—	0	33.3	—	—	33.3	—
***E. faecalis***	100	—	—	—	—	—	33.3	0	—	—	—	0	0	—	—	0	—

AMP: ampicillin; CIP: ciproﬂoxacin; DA: clindamycin; DAP: daptomycin; E: erythromycin; FOS: fosfomycin; GM: gentamicin; LNZ: linezolid; MXF: moxifloxacin; OX: oxacillin; TEC: teicoplanin; TET: tetracycline; TIG: tigecycline; PG: penicillin G; RIF: rifampicin; VA: vancomycin; SXT: trimethoprim/Sulfamethoxazole.

**Table 4:** Antibiotic resistance profile of Gram-negative bacteria.

Antibiotic resistance profile of Gram-negative bacteria (%)																	
	AMP	AK	FEP	AMC	ERT	CAZ	CTX	IMP	CIP	TIG	FOS	GM	LEV	MEM	PIP	PIP/TAZ	CO
***A. baumannii***	100	100	0	0	100	0	0	100	100	0	0	100	100	100	0	0	0
***E. coli***	100	50	50	50	25	50	50	25	50	0	0	25	50	0	50	50	0
***K. pneumoniae***	63.6	18.2	100	72.7	63.6	72.7	81.8	54.6	0	45.4	36.4	54.6	72.7	63.6	91.0	63.6	0
***P. aeruginosa***	0	0	7.7	38.5	15.4	7.7	0	23.1	0	0	61.5	15.4	30.8	15.4	15.4	7.7	0
***K. aerogenes***	100	0	0	100	0	0	0	0	0	0	0	0	0	0	0	0	0
***B. cepacia***	—	—	—	100	—	—	—	0	—	—	—	—	100	0	—	—	—
***P. mirabilis***	66.6	0	66.6	66.6	0	66.6	66.6	0	66.6	100	0	66.6	66.6	0	66.6	0	100

AMP: ampicillin; AK: amikacin; FEP: cefepime; AMC: amoxicillin/clavulanic acid; ERT: ertapenem; CAZ: ceftazidime; CTX: cefotaxime; IMP: imipenem; CIP: ciproﬂoxacin; TIG: tigecycline; FOS: fosfomycin; GM: gentamicin; LEV: levofloxacin; MEM: meropenem; PIP: piperacillin; PIP/TAZ: piperacillin/tazobactam; CO: colistin.

## Data Availability

No data were used to support this study.
